# LncRNA DANCR restrained the survival of *mycobacterium tuberculosis* H37Ra by sponging miR-1301-3p/miR-5194

**DOI:** 10.3389/fmicb.2023.1119629

**Published:** 2023-04-13

**Authors:** Yuliang Qu, Dan Jiang, Minjuan Liu, Hongxia Wang, Tao Xu, Haijin Zhou, Minlan Huang, Weitong Shu, Guangxian Xu

**Affiliations:** ^1^The First Dongguan Affiliated Hospital, Guangdong Provincial Key Laboratory of Medical Molecular Diagnostics, Guangdong Medical University, Dongguan, Guangdong, China; ^2^School of Clinical Medicine, Ningxia Medical University, Yinchuan, Ningxia, China

**Keywords:** DANCR, miRNAs, autophagy, MTB, ceRNA

## Abstract

Tuberculosis is a worldwide contagion caused by *Mycobacterium tuberculosis* (MTB). MTB is characterized by intracellular parasitism and is semi-dormant inside host cells. The persistent inflammation caused by MTB can form a granuloma in lesion regions and intensify the latency of bacteria. In recent years, several studies have proven that long non-coding RNAs (lncRNAs) play critical roles in modulating autophagy. In our study, the Gene Expression Omnibus (GEO) databases were searched for lncRNAs that are associated with tuberculosis. We found that lncRNA differentiation antagonizing non-protein coding RNA (DANCR) increased in the peripheral blood samples collected from 54 pulmonary tuberculosis patients compared to 23 healthy donors. By constructing DANCR overexpression cells, we analyzed the possible cellular function of DANCR. After analyzing our experiments, it was found that the data revealed that upregulation of DANCR facilitated the expression of signal transducer and activator of transcription 3, autophagy-related 4D cysteine peptides, autophagy-related 5, Ras homolog enriched in the brain, and microtubule-associated protein 1A/1B light chain 3 (STAT3, ATG4D, ATG5, RHEB, and LC3, respectively) by sponging miR-1301-3p and miR-5194. Immunofluorescence analysis indicated that DANCR played a positive role in both autophagosome formation and fusion of autolysosomes in macrophages. The colony-forming unit (CFU) assay data also showed that the cells overexpressing DANCR were more efficient in eliminating the intracellular H37Ra strain. Consequently, these data suggest that DANCR restrained intracellular survival of *M*. *tuberculosis* by promoting autophagy *via* miR-1301-3p and miR-5194.

## 1. Introduction

Tuberculosis is a worldwide zoonotic infectious disease caused by *Mycobacterium tuberculosis* (MTB) and seriously threatens human health and endangers public health. Reports show that 2 billion people in the world have been infected with *M. tuberculosis*, with approximately 10% of these individuals developing active tuberculosis (Rohini and Srikumar, 2013). *M. tuberculosis* invades the human body in a variety of ways and damages multiple organs (Gopalakrishnan et al., 2019). The pathogenicity of *M. tuberculosis* mainly depends on a cellular immune response triggered by bacterial proteins, polysaccharides, and lipids. Furthermore, *M. tuberculosis* is characterized by intracellular parasitism and is semi-dormant inside host cells (macrophages, monocytes, and neutrophils) for a long time. These intracellular bacteria lead to persistent inflammation at the infection site. Over time, destruction, repair, proliferation, and necrosis occur simultaneously in lesion regions due to immunoreaction. Eventually, a granuloma (consisting of epithelioid cells, macrophages, lymphocytes, and Langhans cells) can form and intensify the latency of *M. tuberculosis* (Fratti et al., 2003).

Macrophages and phagocytes can counteract the invasion of pathogens through the activation of the effector molecules of the innate immune signal pathway (Ehrt and Schnappinger, 2009); therefore, innate immunity is the body’s first line of defence against *M. tuberculosis*. Among all the defence responses of the innate immune system, macroautophagy (referred to as autophagy) is vital for resisting pathogenic microorganisms. As the name suggests, autophagy is a widely existing and conserved biological phenomenon occurring in the cell; its functions consist of resisting pathogens, maintaining intracellular environmental homeostasis, material circulation, and energy metabolism (Deretic et al., 2013; Nakamura and Yoshimori, 2017; Siqueira et al., 2018; Galluzzi and Green, 2019). The basic process of autophagy includes several stages: (1) the initiation and extension of a cytoplasmic membrane-like structure, (2) the closure of the plasma membrane, (3) the formation of a vesicular autophagosome, and (4) the fusion and maturation of autophagosomes and lysosomes. Thus, it can be seen that autophagy is a dynamic process and presents multiple stages. In other words, autophagy is a precise instrument that is jointly regulated by diverse gene components. Unfortunately, this type of process provides chances for *M. tuberculosis* to manipulate autophagy. Studies have revealed that once innate immune cells are phagocytized by *M. tuberculosis*, the proton pump on the cell membrane decreases due to the influence of the bacterial surface proteins, resulting in the reduction of H^+^ influx and a less acidic environment, which provides a suitable environment for *M. tuberculosis* parasitization (Khan et al., 2019). Another study showed that after invading the human body, *M. tuberculosis* leads to a fat metabolism disorder in macrophages, and the abnormally increased lipid droplet ingestion by autophagosomes was found to co-locate with *M. tuberculosi*s and become the nutrient source for its long-term parasitism (Ouimet et al., 2016). The *M. tuberculosis* bacterial antigen was found to activate different Toll-like receptors (TLRs) and their downstream assembly proteins and multiple signaling pathways, such as the mammalian target of rapamycin, nuclear factor kappa beta, mitogen-activated protein kinases, and phosphoinositide 3 kinase/protein kinase B (mTOR, NF-κB, MAPK, and PI3K/Akt, respectively), by which the process of autophagy was further modulated (Bai et al., 2014; Lai et al., 2016; Liu et al., 2016).

*Mycobacterium tuberculosis* infection also induces the abnormal expression of many non-coding RNA molecules such as microRNAs (miRNA), long non-coding RNA (lncRNA), and circular RNA (circRNA). LncRNAs are RNAs without protein-coding capabilities and have lengths of more than 200 nucleotides. Evidence has shown that lncRNAs are widely involved in physiological and pathological functions, especially in tuberculosis. For instance, the expression level of lncRNA SNHG15 in patients with spinal tuberculosis is significantly increased (Liu et al., 2019). The quantity of lncRNA NEAT1 is significantly elevated in peripheral blood mononuclear cells (PBMCs) isolated from tuberculosis patients and *M. tuberculosis*-infected THP-1 cells (Huang et al., 2018). LncRNA CASC8 polymorphism was proved to be consistent with the trend of increased risk of tuberculosis, suggesting the biomarker potential of CASC8 for tuberculosis clinical progression (Liu et al., 2020). As lncRNAs are extensively involved in the regulation of many genes, many examples of autophagy manipulation by lncRNAs have been observed. For example, lncRNA GAS5 was found to enhance the expression of target genes autophagy-related 5 and 12 (ATG5/ATG12), as well as promote autophagy by competitively adsorbing endogenous miR-181c-5p and 1192 (Xu et al., 2021). LncRNA activation *via* transforming growth factor beta (lncRNA ATB) was shown to boost ATG5 mRNA levels *via* the activation of the yes-associated protein (YAP) and facilitate autophagy in hepatocellular carcinoma cells (Wang et al., 2019). LncRNA functional intergenic repeating RNA element (FIRRE) enhances autophagy by maintaining the stability of beclin 1 (BECN1) mRNA by interacting with polypyrimidine tract-binding protein 1 (PTBP1) (Wang et al., 2022). Based on the previously mentioned studies, it is logical to conclude that lncRNAs play not only crucial roles in *M. tuberculosis* infection but also are an important link between the innate immune system and autophagy regulation, which is worthy of in-depth study.

In this study, we found a high expression of lncRNA differentiation antagonizing non-protein coding RNA (DANCR) in PBMCs isolated from pulmonary tuberculosis patients. Afterward, we constructed a monocyte cell line, THP-1, that stably overexpressed DANCR and further evaluated the molecular function of DANCR using a nucleotide microarray analysis. Through multiple experiments, we demonstrated that DANCR induced autophagy and restrained *M. tuberculosis* survival within macrophages *via* sponging miR-1301-3p and miR-5194.

## 2. Results

### 2.1. DANCR expressed abnormally in tuberculosis patients and H37Ra-infected cells

**Figure 1:**
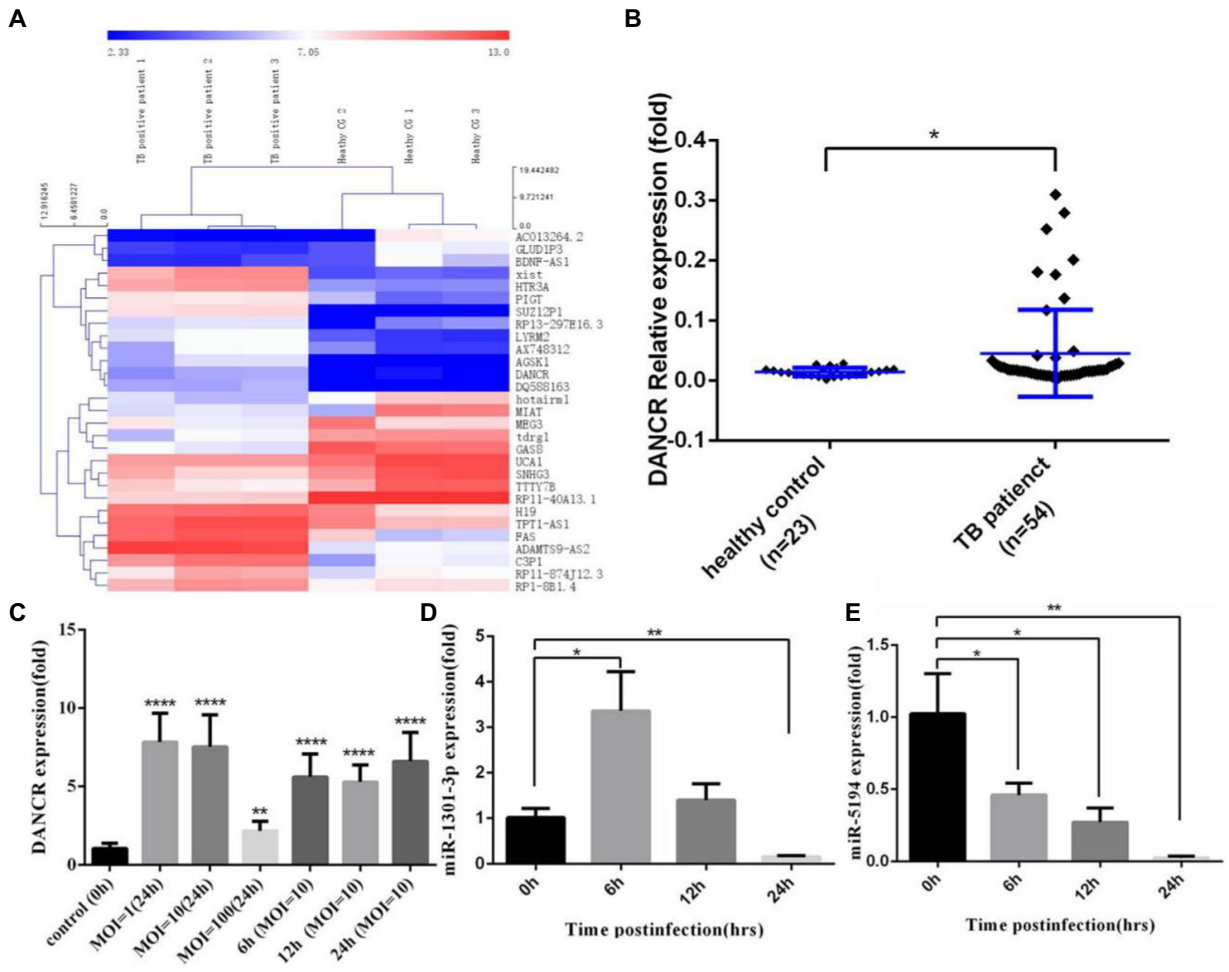
DANCR expressed abnormally in tuberculosis patients and H37Ra-infected cells. **(A)** Differential expression of lncRNAs in tuberculosis patients was acquired from the Gene Expression Omnibus (GEO) database with GEO2R comparison (fold change ≥ 2.0, *p* value ≤ 0.05) and visualized *via* cluster analysis. **(B)** The peripheral blood mononuclear cells (PBMCs) from tuberculosis patients and healthy donors were isolated, and the level of DANCR was detected by real-time reverse transcript polymerase chain reaction (qRT-PCR). **(C)** The monocyte cell line, THP-1 cells were stimulated with PMA before infecting with H37Ra at the MOI of 1, 10, and 100 for 24 h. To explore the variation trend of DANCR, the cells were infected with H37Ra at the MOI of 10 and harvested at indicated time post-infection. The expression of DANCR was determined by qRT-PCR. **(D,E)** THP-1 cells were treated with PMA for 24 h and infected with H37Ra at 10 times MOI. The expression levels of miR-1301-3p **(D)** and miR-5194 **(E)** at different times post-infection were measured by qRT-PCR. Data represent the mean ± standard deviation (SD) from three replications. A two-tailed Student’s *t*-test was used for statistical analysis. *p < 0.05, **p < 0.01, and ****p < 0.0001.

To identify the lncRNAs responsible for MTB-induced autophagy, we first explored the Gene Expression Omnibus (GEO) database and found a project involving active pulmonary tuberculosis patients (He et al., 2017). Using the GEO2R online comparison tool, we profiled the differentially expressed lncRNAs between pulmonary tuberculosis patients and healthy donors (Figure 1A). Some of these genes, such as X-inactive specific transcript (Xist), have been confirmed to be associated with autophagy (Sun et al., 2017; Yao et al., 2020). We found a higher expression of DANCR in tuberculosis patients compared to healthy controls, suggesting that DANCR might be associated with inflammation induced by *M. tuberculosis* infection. To confirm this hypothesis, we collected the peripheral blood from 54 cases of pulmonary tuberculosis patients and 23 cases of healthy donors and detected the expression of DANCR in PBMCs using the qRT-PCR method (Figure 1B). The results showed that the expression levels of DANCR in tuberculosis patients were higher than in healthy controls (*p* = 0.0457), indicating a correlation between DANCR and *M. tuberculosis* infection. Then, we infected THP-1 cells with H37Ra and measured DANCR levels at different multiplicities of infection (MOI = 1, 10, 100) at 24 h (Figure 1C), and the DANCR levels at several times of post-infection with an MOI of 10 were also detected. The results showed that H37Ra infection caused a significant upregulation of DANCR with different MOI. Meanwhile, we also observed the increase of DANCR at an early stage of infection (6 h post-infection). Based on the interaction analysis of lncRNAs and miRNAs (mentioned later), we further explored the variation of miR-1301-3p and miR-5194 in infected cells with an MOI of 10. We noted that miR-1301-3p increased initially and then decreased to a lower level (Figure 1D), whereas miR-5194 declined continuously after H37Ra infection (Figure 1E). These results suggest that DANCR, miR-1301-3p, and miR-5194 may play a role in macrophages against early-stage infection with *M. tuberculosis*.

### 2.2. DANCR interacts with miR-1301-3p and miR-5194

**Figure 2:**
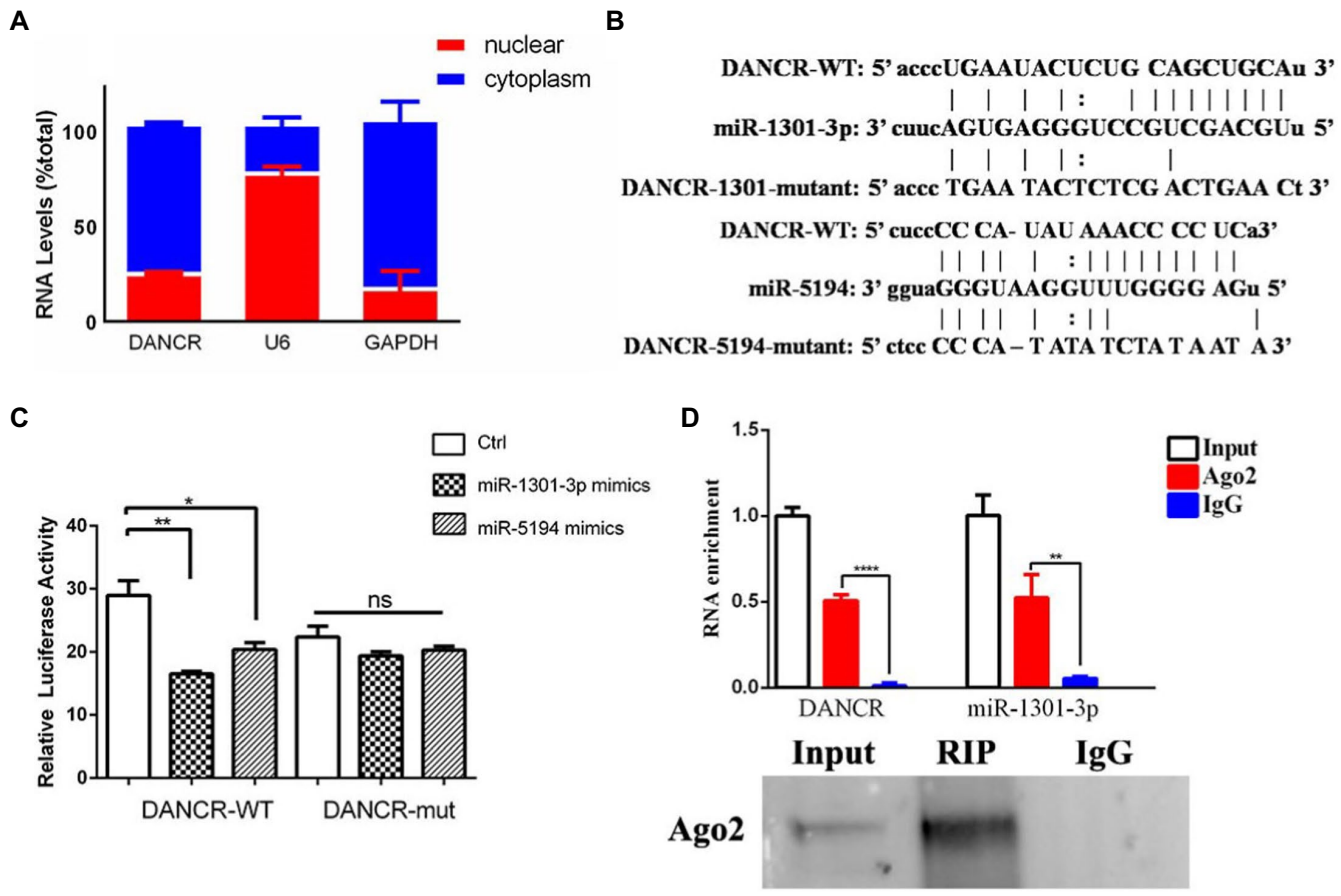
DANCR physically interacted with miR-1301-3p. **(A)** The cytoplasmic and nuclear RNA of PMA-stimulated THP-1 cells were isolated, and the amount of DANCR in different components was measured *via* qRT-PCR. U6 and glyceraldehyde 3 phosphate dehydrogenase (GAPDH) was used as reference genes in the nucleus and the cytoplasm, respectively. **(B)** The sequence of microRNA recognition element (MRE) regions within wild-type DANCR was acquired through Starbase, and the binding sites of miR-1301-3p/miR-5194 were mutant artificially. **(C)** Human embryonic kidney (HEK293) cells were co-transfected with mimics of miR-1301-3p/miR-5194 and the pmiR-GLO vector containing wild-type DANCR plasmid (DANCR-WT) or mutant DANCR plasmid (DANCR-mut). The cells were harvested for measuring luciferase activity after 48 h. **(D)** The PMA-stimulated THP-1 cells were lysed and precipitated by magnetic beads coupled with Argonaute 2 (Ago2) antibodies, and the precipitates were subsequently detected by using qRT-PCR (upper panel) and Western blotting (bottom panel). Normal IgG was used as a negative control. Data represent the mean ± standard deviation (SD) from three replications. *p < 0.05, **p < 0.01, and ****p < 0.0001.

Given that the lncRNA sponging of miRNAs occurs in the cytoplasm, we isolated cytoplasmic and nuclear RNA from PMA-stimulated THP-1 cells and determined DANCR levels among different cellular components (Figure 2A). The results showed that DANCR was mainly distributed in the cytoplasm, and only a small amount was present in the nucleus. U6 and GAPDH were used as reference genes in the nucleus and cytoplasm, respectively. We, then, analyzed the DANCR sequences using the Starbase database to acquire the binding sites of miRNA response elements (MREs). As shown in Figure 2B, we found DANCR sequences that contained seed regions of miR-1301-3p and miR-5194. We further mutated the binding sites to construct a luciferase report vector for subsequent experiments (Figure 2B). Next, we transfected different combinations of vectors and miRNA mimics into human embryonic kidney cells (HEK293T) and measured luciferase activity (Figure 2C). Luciferase activity decreased significantly in the group co-transfected with the DANCR-wild type (WT) vector and miR-1301-3p/miR-5194 mimics. Once the complementary sequence was destroyed, the inhibitory capabilities of miR-1301-3p/miR-5194 toward luciferase activity no longer existed, which proved the presence of an interaction between DANCR and miR-1301-3p/miR-5194. After that, we continued to explore whether DANCR, miR-1301-3p, and miR-5194 could form an RNA-induced silencing complex (RISC). We selected Argonaute-2 (Ago2), one of the core components of RISC, to capture the protein–RNA complex. The PMA-stimulated THP-1 cells were lysed, and the protein–RNA complex was obtained using magnetic beads coupled with Ago2 antibodies, and the precipitates were subsequently obtained (Figure 2D). The results of the qRT-PCR test showed abundant DANCR and miR-1301-3p detected in the anti-Ago2 group compared to that in the normal IgG group. The results of immunoprecipitation were further confirmed by Western blotting. These results suggest that DANCR physically interacts with miR-1301-3p and enters RISC to execute subsequent functions. The above findings indicate that DANCR very likely regulates genes downstream of miR-1301-3p *via* a competing endogenous RNA (ceRNA) mechanism. Despite the weaker interaction between DANCR and miR-5194, we still utilized miR-5194 in the following experiment as an endogenous reference.

### 2.3. Bioinformatics analysis of DANCR overexpression

**Figure 3:**
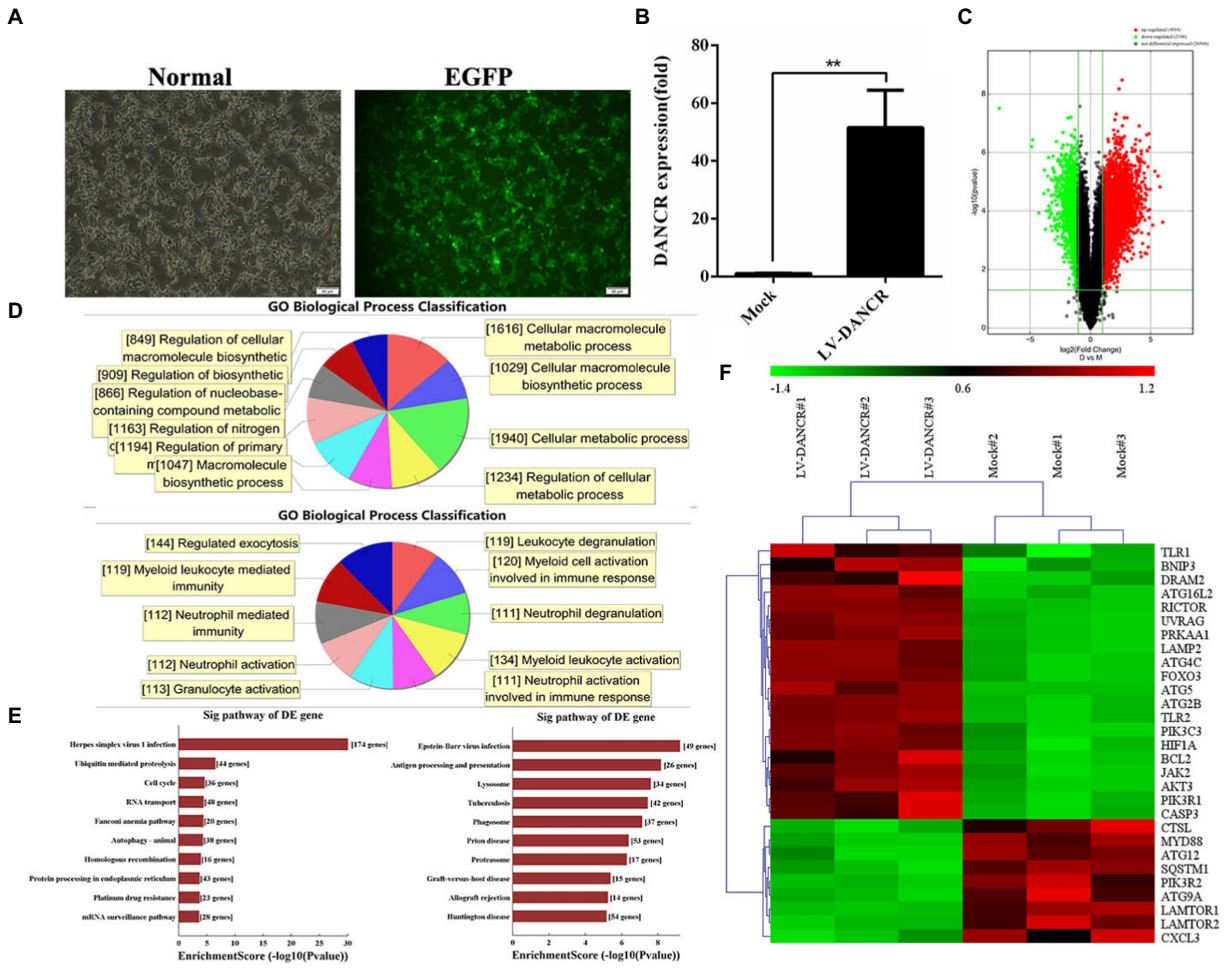
Bioinformatics analysis of DANCR overexpression. **(A)** THP-1 cells were transfected with lentivirus encoding DANCR overexpression plasmid for 3 days and further treated with puromycin to construct lentivirus (LV)-DANCR cells (green fluorescent protein-positive). The images showed the PMA-treated LV-DANCR cells at a magnification of 200. **(B)** The quantity of DANCR in LV-DANCR cells was confirmed *via* qRT-PCR. Data represent the mean ± SD from three replications. A two-tailed Student’s *t*-test was used for statistical analysis. **p < 0.001. **(C)** The LV-DANCR cells were detected by whole human genome oligo microarray, and the differential probe signal of mRNAs was screened using value of p and the FDR method and exhibited as volcano map (fold change ≥ 2.0, value of p ≤ 0.05, FDR ≤ 0.05). The red dots represented the elevated genes, and the green dots stood for descending genes. **(D)** The differential mRNAs in LV-DANCR cells compared to normal THP-1 cells were identified and annotated with gene ontology (GO). The most enriched biological process terms of elevated mRNAs (upper panel) and decreasing mRNAs (bottom panel) were drew by computation. **(E)** A Kyoto Encyclopedia of Genes and Genomes (KEGG) analysis was performed to enrich differential expressed mRNAs in LV-DANCR cells. The elevated mRNAs (left) and the decreased mRNAs (right) were grouped with identical pathway terms and sorted by numbers. **(F)** The genes that were highly associated with autophagy were identified by GO annotation and KEGG analysis in advance and then clustered for revealing similar biological properties in two groups. (lane 1–3: LV-DANCR cells, lane 4–6: normal THP-1 cells).

To further clarify the molecular function of DANCR *in vitro*, we constructed a THP-1 cell line that expressed excessive DANCR (hereinafter referred to as LV-DANCR cells) through lentivirus infection and puromycin screening. The positive rate of LV-DANCR cells was confirmed by examining the enhanced green fluorescent protein (EGFP) under microscopy (Figure 3A), and the DANCR level was quantified with qRT-PCR (Figure 3B). After cultivating a large number of LV-DANCR cells, we measured oligonucleotide signals by microarray hybridization, and the differentially expressed probes were acquired through fold change (> 1.0) and value of p(< 0.05) filtering (Figure 3C). Compared with the normal THP-1 cells, 4,014 probe signals (3,242 mRNAs) increased in the LV-DANCR cells, whereas 2,186 (1,933 mRNAs) probe signals decreased. After gene ontology (GO) annotation of the above differentially expressed mRNA, we observed that the elevated genes were mainly involved in cell metabolism (1940), cell macromolecule metabolism (1616), cell metabolic process regulation (i.e., the regulation of cellular metabolic processes; 1234), and other functions related to the synthesis and metabolism of biological macromolecules, which indicated that DANCR might participate in the biological process dominated by metabolism and molecular synthesis through these genes (Figure 3D, upper panel). On the other hand, the functions of descending genes were involved in myeloid cell activation (134), myeloid cell activation activated in immune response (120), myeloid cell-mediated immune response (119), granulocyte degranulation (119), and other biological processes related to granulocyte immune function, which indicates that DANCR might have a role in the immune function of myeloid cells (Figure 3D, bottom panel). Moreover, the data of Kyoto Encyclopedia of Genes and Genomes (KEGG) pathway analysis revealed that the elevated mRNAs (Figure 3E, upper panel) were responsible for herpes simplex virus 1 infection (174), ubiquitin-mediated protein hydrolysis (44), cell cycle (36), RNA transport (48), and autophagy (38). Meanwhile, the reduced mRNAs in LV-DANCR cells (Figure 3E, bottom panel) were found to be involved in Epstein–Barr virus infection (48), antigen processing and presentation (26), tuberculosis (42), lysosomes (34), autophagosomes (37), prion disease (53), and other pathways. The data of bioinformatics clearly showed that DANCR was closely related to the infection process dominated by pathogen invasion. In addition, we discovered that DANCR overexpression altered many autophagy-related genes after performing a cluster analysis (Figure 3F). Based on these findings, we presume that DANCR might be a crucial node in mediating pathogen infection and autophagy regulation.

### 2.4. miR-1301-3p and miR-5194 inhibit autophagy

**Figure 4:**
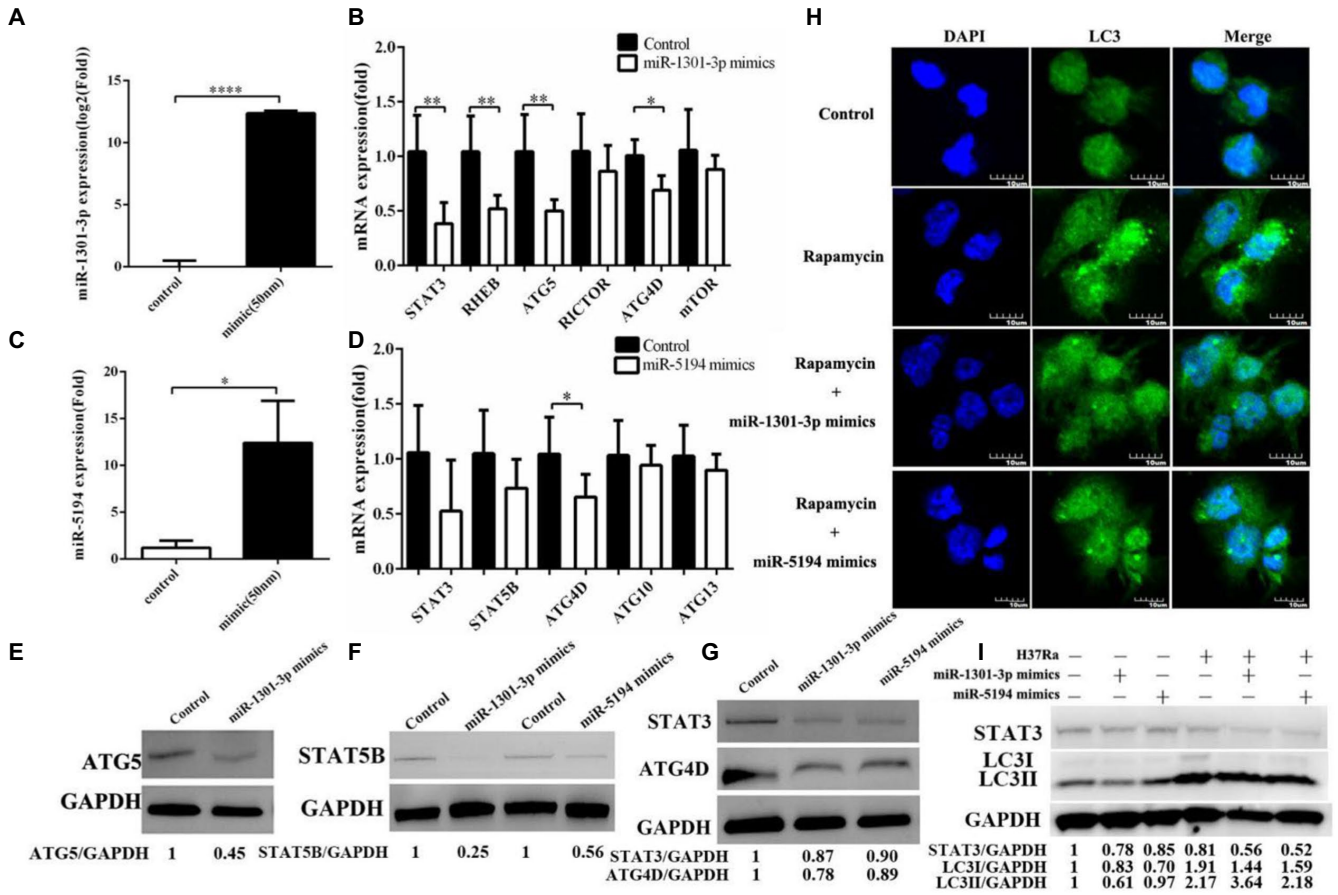
miR-1301-3p/miR-5194 inhibited autophagy. **(A–D)** THP-1 cells were transfected with miR-1301-3p mimics **(A)** or miR-5194 mimics **(C)** for 24 h, and mRNA levels were measured using qRT-PCR **(B)** potential targets of miR-1301-3p, **(D)** potential targets of miR-5194). **(E–G)** THP-1 cells were transfected with mimics of miR-1301-3p/miR-5194 for 48 h and proteins associated with autophagy-related 5 (ATG5) **(E)**, signal transducer, and activating transcription 5B (STAT5B), **(F)** STAT3 and autophagy-related 4D cysteine proteinase (ATG4D) **(G)** were detected by Western blotting. **(H)** The THP-1 cells were stimulated with PMA and then transfected with miRNA invalid fragments (control) or miR-1301-3p/miR-5194 mimic for 24 h. Afterward, rapamycin was added for 2 h. The microtubule-associated protein 1A/1B light chain 3 (LC3) puncta were tracked with antibodies followed by fluorescein isothiocyanate (FITC)-conjugated IgG. The pictures were captured at a magnification of 600. **(I)** The THP-1 cells were stimulated with PMA and transfected with miR-1301-3p/miR-5194 mimics for 24 h and further infected with H37Ra for 24 h. Both STAT3 and LC3 proteins were measured using Western blotting. Data represent the mean ± SD from three replications. A two-tailed Student’s *t*-test was used for statistical analysis. *p < 0.05, **p < 0.01, and ****p < 0.0001.

Considering the physical interaction between DANCR and miR-1301-3p, we, next, explored the genes that are downstream of miR-1301-3p. We first transfected miR-1301-3p or miR-5194 mimics into THP-1 cells and verified the efficiency of expression *via* qRT-PCR (Figures 4A,C). Afterward, we obtained several potential targets (STAT3, STAT5B, ATG4D, ATG5, ATG10, ATG13, RICTOR, RHEB, and mTOR) of miR-1301-3p/miR-5194 through miRDB, miRbase, and miRWalk databases. Then, the mRNA levels of the above targets were determined in miR-1301-3p or miR-5194 mimics transfected cells (Figures 4B,D). As a result of qRT-PCR, the overexpression of miR-1301-3p was shown to inhibit STAT3, RHEB, ATG5, and ATG4D, whereas excess miR-5194 impaired the expression of ATG4D. In addition, the results of Western blotting were consistent with those of qRT-PCR (Figures 4E–G). As for decreasing the protein levels of STAT3 and STAT5B in the miR-5194 mimics group, the results suggested that miR-5194 might fine-tune the translation of STAT3 and STAT5B. As the above genes played diverse or even opposite roles in the regulation of autophagy, we utilized miR-1301-3p/miR-5194 mimics plus rapamycin to assess the level of autophagy (Figure 4H). As shown in Figure 4H, compared to miRNA invalid fragments plus rapamycin (control), extensive LC3 puncta appeared in the THP-1 cells treated with rapamycin. This trend was reversed when either miR-1301-3p or miR-5194 increased. Subsequently, we infected THP-1 cells with H37Ra and detected the expression of STAT3 and LC3 proteins *via* Western blotting (Figure 4I). The expression of STAT3 was depressed, which was consistent with Figure 4G. The results also showed that miR-1301-3p overexpression led to a significant reduction in both LC3-I and LC3-II proteins. With H37Ra infection, miR-1301-3p slightly decreased the amount of LC3-I protein and failed to block the conversion of LC3-I to LC3-II. These findings validate the role of ATG5 both in autophagosome formation and lysosome maturation. The decreased LC3-I levels caused by miR-5194 mimics were most likely due to the processing ability of the ATG4 family toward proLC3. This evidence implies that both miR-1301-3p and miR-5194 produce an inhibitory effect on autophagy.

### 2.5. DANCR induced autophagy by sponging miR-1301-3p/miR-5194

**Figure 5:**
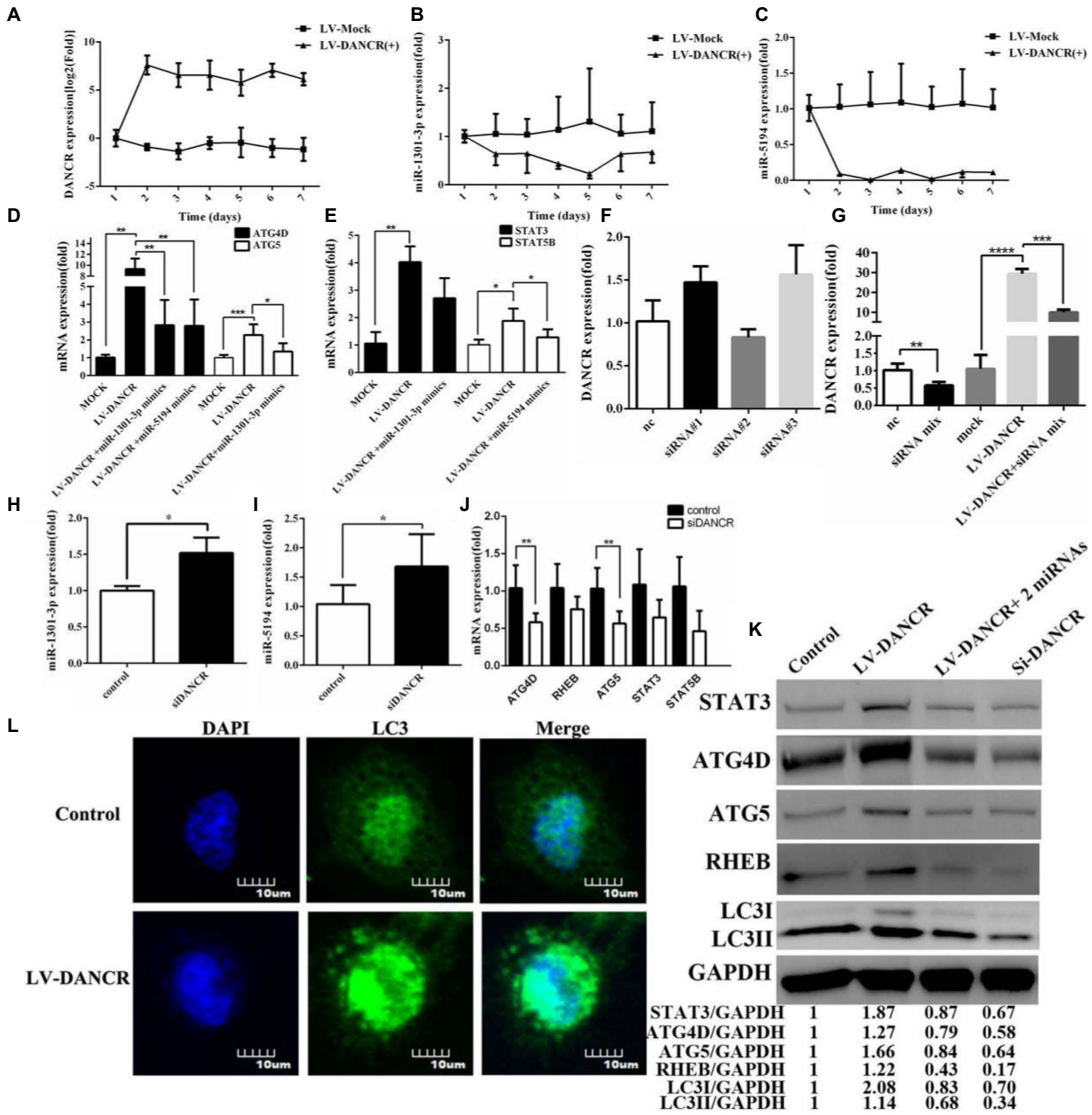
DANCR induced autophagy by sponging miR-1301-3p/miR-5194. **(A–C)** The PMA pretreated THP-1 cells were transfected with lentivirus encoding DANCR for 3 days. After that, the cells were harvested every 24 h for a week. The expression of DANCR **(A)**, miR-1301-3p **(B)**, and miR-5194 **(C)** on each day was determined by qRT-PCR. **(D)** The LV-DANCR cells were transfected with or without miR-1301-3p mimics for 24 h, and mRNA levels of ATG4D and ATG5 were quantified by qRT-PCR. **(E)** The LV-DANCR cells were transfected with or without miR-5194 mimics, and the expression of STAT3 and STAT5B was detected *via* qRT-PCR. **(F)** Three siRNAs of DANCR were transfected into THP-1 cells, and DANCR levels were tested *via* qRT-PCR. **(G)** Three small interfering RNAs (siRNAs) were mixed intensively and transfected into THP-1 or LV-DANCR cells to evaluate the silencing efficiency of DANCR. **(H–J)** After DANCR was suppressed, the expression of miR-1301-3p **(H)**, miR-5194 **(I)**, and five mRNAs **(J)** was detected *via* qRT-PCR. **(L)** THP-1 cells were transfected with lentivirus encoding DANCR for 3 days, and the LC3 puncta were analyzed through confocal microscopy at a magnification of 600. **(K)** The proteins associated with STAT3, ATG4D, ATG5, RHEB, and LC3 in different groups (lane 1: control, lane 2: LV-DANCR cells, lane 3: LV-DANCR plus mimics with two miRNAs, and lane 4: THP-1 cells transfected with siRNAs mixture were measured by Western blotting. Data represent the mean ± SD. A two-tailed Student’s *t*-test was used for statistical analysis. *p < 0.05, **p < 0.01,***p < 0.001, and ****p < 0.0001.

After identifying the target genes of miR-1301-3p/miR-5194, we investigated whether DANCR regulated these genes through the ceRNA mechanism. We continuously monitored the levels of DANCR, miR-1301-3p, and miR-5194 in THP-1 cells that were newly transfected with lentivirus (Figures 5A–C). Data were acquired over 7 days, which indicated that DANCR increased rapidly and maintained a high level of activity in its plateau phase (Figure 5A). Notably, the expressions of miR-1301-3p (Figure 5B) and miR-5194 (Figure 5C) were constantly suppressed synchronously, which suggested that DANCR sponged both of these miRNAs. Therefore, we, next, detected target gene levels of miR-1301-3p and miR-5194 in LV-DANCR cells *via* qRT-PCR (Figures 5D,E, respectively). The results showed that DANCR overexpression led to a significant promotion in the expressions of ATG4D/ATG5 (Figure 5D) and STAT3/STAT5B (Figure 5E). Notably, we further transfected miR-1301-3p/miR-5194 mimics into LV-DANCR cells and observed a decrease in ATG4D/ATG5, but the levels of these two mRNAs were still higher than in the controls (Figure 5D). In other words, for ATG4D/ATG5, the promotion of DANCR was stronger than the inhibitory effect of miR-1301-3p/miR-5194. Similar results can also be seen for STAT3/STAT5B (Figure 5E). Given that DANCR is mainly distributed in the cytoplasm, we utilized small interfering RNA (siRNA) to construct DANCR-silencing model cells. A total of three different siRNAs for DANCR were synthesized and transfected into THP-1 cells, after which DANCR levels were quantified. As shown in Figure 5F, the three siRNAs minimally inhibited DANCR when administered separately. We transfected THP-1 cells or LV-DANCR cells with a mixture containing the three siRNAs and remeasured the DANCR levels (Figure 5G). Notably, the siRNAs mixture caused a reduction in DANCR levels not only in normal THP-1 cells but also in LV-DANCR cells, a result that suggests co-interference with multiple sites could be an accessible strategy to silence lncRNAs. When DANCR expression decreased, we found that the expressions of miR-1301-3p and miR-5194 increased accordingly (Figures 5H,I, respectively), and the mRNA levels of ATG4D, ATG5, RHEB, STAT3, and STAT5B decreased as long as siRNAs were present (Figure 5J). In addition to LC3 *via* Western blotting, we further examined the proteins associated with the above genes (Figure 5K). The data showed protein levels of ATG4D, ATG5, RHEB, STAT3, and LC3 in LV-DANCR cells were obviously improved. Meanwhile, similar to the siRNA addition (siDANCR) group, the overexpression of miR-1301-3p and miR-5194 led to a slight decrease in those proteins in LV-DANCR cells. Immunofluorescence analysis was performed to explore LC3 puncta formation, and the graphics in Figure 5L showed DANCR overexpression amplified the amount of LC3 puncta, indicating autophagy activation. These findings prove DANCR-induced autophagy by sponging miR-1301-3p and miR-5194.

### 2.6. DANCR restrained the survival of intracellular *Mycobacterium tuberculosis* by inducing autophagy

**Figure 6:**
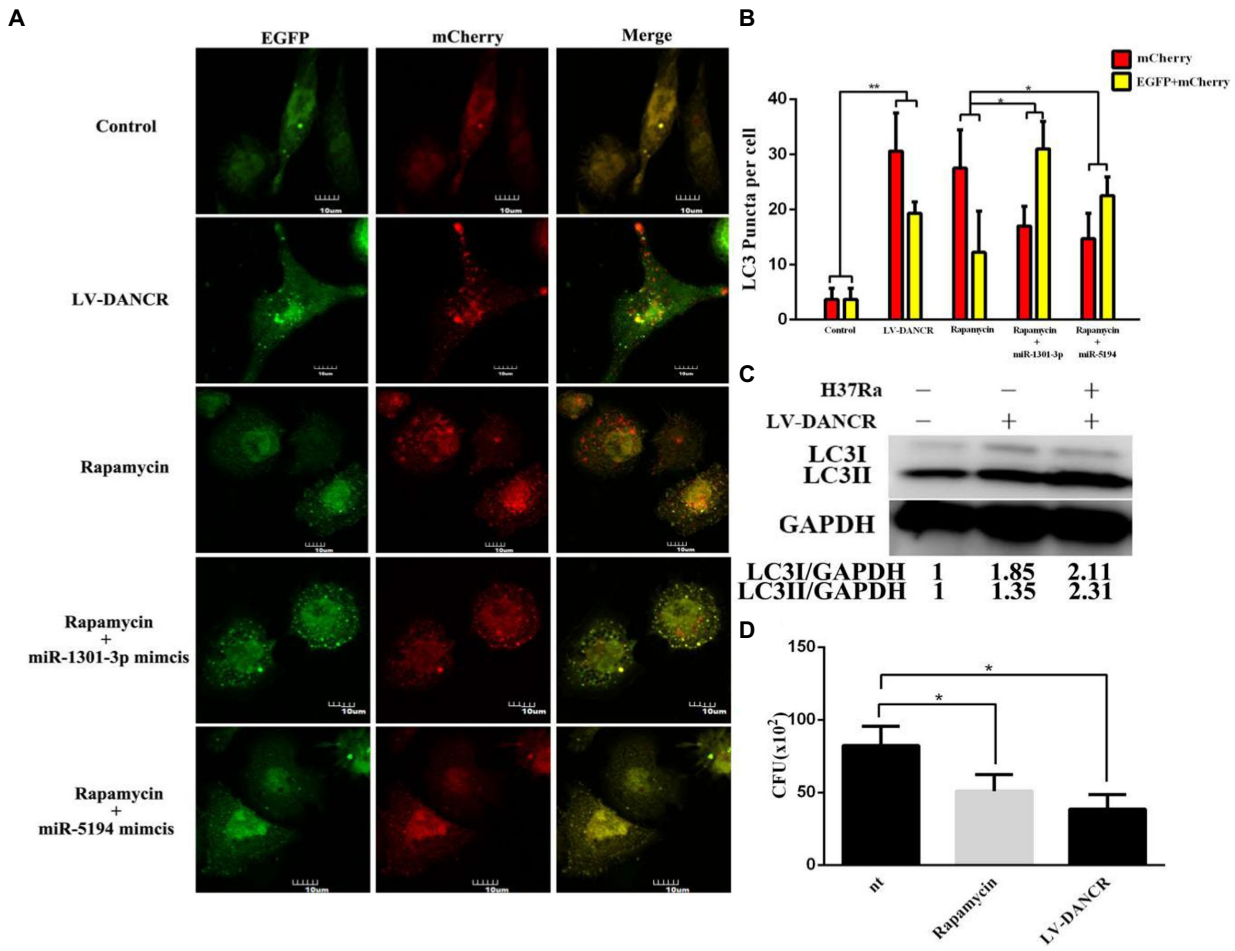
DANCR restrained the survival of intracellular MTB by inducing autophagy. **(A)** Normal THP-1 cells were transfected with lentivirus encoding EGFP-mCherry-LC3 plasmid to construct cellular models for autophagic flux detection. Afterward, the cell models were treated with PMA followed by lentivirus encoding DANCR, rapamycin, and rapamycin plus miR-1301-3p/miR-5194 mimics within 72 h, respectively. The images were captured at the magnification of 600. **(B)** The red or yellow puncta of LC3 in each group were counted from three different fields under the microscope and at least 10 cells. **(C)** The LC3 proteins in normal THP-1(lane 1), LV-DANCR cells (lane 2), and LV-DANCR cells infected with H37Ra (lane 3) were measured by Western blotting. **(D)** Normal THP-1 cells, rapamycin-pretreated THP-1 cells, and LV-DANCR cells were infected with H37Ra for 6 h. The cells were washed with PBS thoroughly to remove the extracellular H37Ra and cultured for another 18 h before being lysed. The cell fragments were inoculated on 7H10 agar plates, and the colony of H37Ra was counted 3 weeks later. Data represent the mean ± SD from three replications. A two-tailed Student’s *t*-test was used for statistical analysis. *p < 0.05 and **p < 0.01.

To determine at which stage DANCR facilitated autophagy, autophagic flux analysis was performed (Figures 6A,B). As a result of immunofluorescence, the LV-DANCR cells showed more yellow or red LC3 dots than the control group, suggesting an increase in the number of autophagosomes and autolysosomes. Rapamycin was used as an autophagy-positive reference. When rapamycin was added to THP-1 cells previously transfected with miR-1301-3p or miR-5194 mimics, the number of green spots (autolysosome) decreased, indicating that the fusion of autophagosome and lysosome was blocked by the miR-1301-3p/miR-5194 mimics. Moreover, we observed greater levels of both LC3-I and LC3-II proteins in LV-DANCR cells than in normal cells and those with H37Ra infection (Figure 6C). The survival of intracellular H37Ra was further explored through a colony-forming unit (CFU) assay (Figure 6D). The results showed that less *M. tuberculosis* was parasitized in LV-DANCR cells compared to normal THP-1 cells and in the rapamycin-treated group, indicating that the long-term overexpression of DANCR was conducive to macrophage activation to remove intracellular H37Ra, which further proved that DANCR restrained intracellular bacteria by promoting autophagy.

## 3. Materials and methods

### 3.1. Diagnosis of TB and clinical samples collection

The procedure of clinical blood sample collection was approved by the ethics committee of Ningxia Medical University (grant number: 2018–026), and signed informed consent was obtained from all subjects. For the pulmonary tuberculosis group, patients fulfilling several conditions were included as follows: (1) individuals met the clinical diagnostic standard of pulmonary tuberculosis and (2) met the condition of smear-positive and/or blood culture-positive tuberculosis. Possible interfering factors due to hepatitis B virus, *Treponema pallidum*, and human immunodeficiency virus were excluded. The age range of 54 (men: 29, women: 25) patients recruited in this study was 18–80 years old. The average age of the patient group is 46 years old. A total of 23 healthy controls were enrolled and consisted of (men: 13, women: 10) physical examination donors younger than 35 years old (average age is 26 years old), without a history of hepatitis B virus, *T. pallidum*, and/or human immunodeficiency virus infection. PBMCs were isolated from the aforementioned blood samples *via* Ficoll centrifugation (Solarbio, China).

### 3.2. Cell culture and transfection

THP-1 cells and HEK-293 T cells were purchased from the Type Culture Collection of the Chinese Academy of Sciences (Shanghai, China). The THP-1 cells were maintained in Roswell Park Memorial Institute (RPMI) 1640 medium (Biological Industries, Israel) containing 15% fetal bovine serum (FBS, Biological Industries, Israel), while HEK-293 T cells were cultured in Dulbecco’s modified Eagle’s medium (DMEM) with 10% FBS. All cells were incubated at 37°C under 5% CO_2_. The THP-1 cells utilized in the experiments were stimulated with phorbol myristate acetate (PMA) for 24 h at the concentration of 100 ng per microliter before further treatment. For cell transfection, the PMA-stimulated THP-1 cells were transfected with 50 nM control or miR-1301-3p/miR-5194 mimic (GenePharma, China), or 50 nM siRNA (GenePharma, China) of DANCR using Lipofectamine 3000 (Invitrogen, United States), according to the manufacturer’s instructions.

### 3.3. Nuclear and cytoplasm fractionation

The nuclear and cytoplasm fractions from THP-1 cells were isolated using a PARIS™ Kit (Invitrogen, United States). For nuclear isolation, 1 × 10^7^ THP-1 cells were harvested and resuspended in the cell fraction buffer for 10 min of lysis. After centrifugation, supernatant and nuclear pellets were separated for RNA extraction using the cell disruption buffer, according to the manufacturer’s instructions.

### 3.4. Bacterial strains and CFU assays

The H37Ra strain was purchased from the Center for Disease Control and Prevention of China (Beijing, China), and bacilli were cultured in Middlebrook 7H9 medium with 10% albumin dextrose catalase (ADC) at 37°C for 3 weeks. For cell infection, the H37Ra strain was collected and disrupted by low-frequency ultrasonic. Bacterial concentrations were calculated *via* absorbance measurement at 600 nm. THP-1 cells were infected at the indicated multiplicity of infection (MOI) for 6 h and then washed with phosphate-buffered saline (PBS). For colony-forming unit (CFU) assays, LV-DANCR cells were infected by H37Ra suspension for 6 h and washed with PBS to remove extracellular bacterium. The infected cells were incubated at 37°C for another 24 h and lysed with 0.2% Triton X-100. Afterward, cell lysates were diluted to a 10-fold serial dilution and inoculated on 7H10 agar plates with oleic acid–albumin–dextrose–catalase (OADC). The plates were incubated at 37°C for 3 weeks after which the colonies were counted.

### 3.5. The whole human genome oligo microarray detecting and bioinformatics analysis

The total RNA of LV-DANCR was isolated and measured by Nanodrops ND-1000. RNA integrity was assessed by agarose gel electrophoresis. All RNA samples were labeled and hybridized according to the Agilent One-Color Microarray-Based Gene Expression Analysis protocol (Agilent Technology, United States). A measure of 100 microliters of hybridization solution was dispensed onto the microarray slide followed by a 17 h incubation at 65°C. Agilent Feature Extraction software and GeneSpring GX v12.1 software package (Agilent Technologies) were used to obtain normalized raw data. The differentially expressed signals were identified through fold change filtering (fold change≥1, value of *p*≤0.05) *via* both value of p (two groups compared with *t*-test) and the obtained false discovery rate (i.e., FDR), according to the Benjamin Hochberg FDR method. All the differentially expressed genes were visualized as the volcano plot *via* the limma (Linear Models for Microarray Data) package, an R programming language of the computer. Gene ontology (GO) and pathway analyses were performed using topGO under the standard enrichment computation method. The potential targets of miR-1301-3p/miR-5194 with a high score were acquired through miRDB,^1^ miRbase,^2^ and miRWalk.^3^

### 3.6. Plasmid construction and luciferase reporter assays

The wild-type or mutant sequence of DANCR was synthesized and cloned into the pmiR-GLO vector (Zoonbio, China) to construct the luciferase reporter plasmid. A measure of 100 nanograms of luciferase reporter plasmid was co-transfected with 50 nM control or miR-1301-3p/miR-5194 mimics into 293 T cells with Lipofectamine 3000 (Invitrogen). The cells were lysed after 48 h and evaluated using the Dual-Luciferase Reporter Assay System (Promega), following the manufacturer’s protocol.

### 3.7. Quantitative real-time PCR

The expression of DANCR, miR-1301-3p, miR-5194, and target mRNAs was detected using real-time polymerase chain reaction (qRT-PCR). Total RNA was produced using the TRIzol reagent (Sigma). The cDNA was synthesized *via* the Revert Aid First Strand cDNA Synthesis Kit (Thermo Fisher Scientific), and qRT-PCR was performed using the SYBR Green PCR Kit (QuantiNava, German). RNU6 (for miRNA measuring) or glyceraldehyde 3 phosphate dehydrogenase (GADPH) for DANCR and mRNA detection, respectively, was used for normalization. The primers used in qRT-PCR are shown in Table 1. The quantity was calculated by using a relative quantity value (2^-△△Ct^) method.

**Table 1:** Primer details of RT-qPCR.

Gene ID	Sequence (5′ to 3′)
DACNR Forward	AATGCAGCTGACCCTTACCC
DANCR Reverse	GGCTTCGGTGTAGCAAGTCT
STAT3 Forward	CTGTGGGAAGAATCACGCCT
STAT3 Reverse	ACATCCTGAAGGTGCTGCTC
STAT5B Forward	GACCAAGTTTGCAGCCACTG
STAT5B Reverse	ATTGCGGGTGTTCTCGTTCT
miR-1301-3p Stem-loop	CTCAACTGGTGTCGTGGAGTCGGCAATTCAGTTGAGGAAGTCAC
miR-1301-3p Forward	ACACTCCAGCTGGGGCTGGGGATGTTGCAGCTGCCTGGGAGT
miR-1301-3p Reverse	TGGTGTCGTGGAGTCG
miR-5194 Stem-loop	CTCAACTGGTGTCGTGGAGTCGGCAATTCAGTTGAGAGCGGCCA
miR-5194 Forward	ACACTCCAGCTGGGGTGAGGGGT TTGGAATG
miR-5194-Reverse	TGGTGTCGTGGAGTCG
RICTOR Forward	AGTGAATCTGTGCCATCGAGT
RICTOR Reverse	AGTAGAGCTGCTGCCAAACC
ATG4D Forward	AGCTCCTCCTCAGCCACA
ATG4D Reverse	GGAGCAGAGGTCGTCCAG
ATG10 Forward	CCCAGCAGGAACATCCAATA
ATG10 Reverse	AGGCTCAGCCATGATGTGAT
RHEB Forward	ATGCCTCAGTCCAAGTCCCGGAAG
RHEB Reverse	TCACATCACCGAGCACGAAGA
U6 Forward	CTCGCTTCGGCAGCACA
U6 Reverse	AACGCTTCACGAATTTGCGT
ATG5 Forward	AAAGATGTGCTTCGAGATGTGT
ATG5 Reverse	CACTTTGTCAGTTACCAACGTCA
ATG16L1 Forward	CAGAGCAGCTACTAAGCGACT
ATG16L1 Reverse	AAAAGGGGAGATTCGGACAGA
ATG13 Forward	ATAAGAATGCGGCCGCATGGAAACTGA
ATG13 Reverse	CTAGTCTAGATTACTGCAGGGTTTCCAC
GAPDH Forward	GGTCTCCTCTGACTTCAACA
GAPDH Reverse	GTGAGGGTCTCTCTCTTCCT

### 3.8. Western blotting

All proteins were loaded onto a 4–12% sodium dodecyl sulfate-polyacrylamide gel electrophoresis (SDS-PAGE) gel and transferred to a polyvinylidene difluoride (PVDF) (Millipore, United States) membrane. PVDF membranes were blocked with 5% bovine serum albumin (BSA) and incubated overnight with the corresponding antibodies (purchased from Abcam, United States). Then, the membranes were washed with Tris-Buffered saline with Tween (TBST) and further incubated with horseradish peroxidase (HRP)-conjugated goat IgG for 1 h. Eventually, protein bands were analyzed by using the electrochemiluminescence (ECL) reagent (Thermo Fisher Scientific, United States). The intensity quantitation of Western blotting was analyzed by Image J software.

### 3.9. Confocal microscopy

The THP-1 cells were treated with phorbol myristate acetate (PMA) and fixed with 4% paraformaldehyde followed by fixation with 0.2% Triton X-100. After blocking with 5% BSA, cells were incubated with the LC3 antibody and fluorescein isothiocyanate (FITC)-conjugated goat IgG. Images were captured by Olympus DSU confocal microscope under a magnification of 600. At least 10 cells from triple cross-sectioned per group were counted by blinded researchers.

### 3.10. Lentivirus infection system and autophagic flux detection

The lentivirus encoding DANCR was constructed by GeneChem (Shanghai, China). THP-1 cells were infected with lentivirus at an MOI of 30 for 3 days to construct the primary LV-DANCR cells. Next, the primary cells were further treated with puromycin (1 μg/mL) for 7 days to eliminate negative ones. The pictures of positive LV-DANCR cells were captured by a fluorescent microscope at a magnification of 200. For autophagic flux detection, THP-1 cells were infected with lentivirus encoding an enhanced green fluorescent protein (EGFP), red fluorescent protein (mCherry), as well as LC3 plasmid (referred to as EGFP-mCherry-LC3 plasmid), to construct stable cell models before further testing. The cell models are next treated as shown in Figure 6A.

### 3.11. RNA immunoprecipitation

RNA immunoprecipitation (RIP) was performed using the RNA Immunoprecipitation Kit (GeneSeed, China), according to the manufacturer’s instructions. Ago2 antibody and normal IgG were used for RIP experiments. Co-precipitated RNAs were examined by qRT-PCR while co-precipitated proteins were measured by Western blotting.

## 4. Discussion

In recent years, mounting evidence has indicated that lncRNAs are closely related to *M. tuberculosis* infection. For example, it was reported that Bacillus Calmette-Guerin (BCG) infection increased the expression of myocardial infarction-associated transcript (MIAT) in addition to lincRNA-Cox2 in human and mouse macrophages (Jiang et al., 2021; Xu et al., 2021). In addition, emerging studies have indicated that lncRNAs might act as ceRNAs to regulate gene expression through the competitive binding of MREs, which would represent a new post-transcriptional regulatory mechanism (Mercer et al., 2009). DANCR is a novel lncRNA identified in 4q12.5 (Tong et al., 2015), and reports on DANCR have focused on its effect as an oncogene (Pan et al., 2020; Zhang et al., 2021). In this study, we discovered that DANCR was elevated in PBMCs of pulmonary tuberculosis patients, which showed some correlation between DANCR and tuberculosis. As described earlier, non-coding RNAs were proven to be a possible diagnostic marker of tuberculosis because of their extreme sensitivity. To improve sensitivity and specificity, it is necessary to utilize both lncRNA and miRNAs for molecular diagnosis. In our project, we observed that DANCR tended to restrain endogenous miR-1301-3p and miR-5194. This finding may be favorable for developing new molecular tools for tuberculosis diagnosis.

After constructing the THP-1 cells overexpressing DANCR, we also mapped the potential function of DANCR *via* nucleotide microarray detection. Noticeably, the data suggest that DANCR is closely related to the modification of micro-proteins (e.g., ubiquitin), which will be explored in our subsequent research. In the present study, we examined the role of DANCR in the innate immune system as a promoter of autophagy. Furthermore, we also confirmed the “sponge/decoy” capability of DANCR. DANCR was found to regulate the expression of several genes (STAT3, STAT5B, RHEB, ATG4D, and ATG5) *via* miR-1301-3p and miR-5194 at post-transcriptional levels. Interestingly, these genes control autophagy in different ways. For example, nuclear STAT3 regulates autophagy *via* the beat cell lymphoma (Bcl2) family members (Shen et al., 2012), whereas cytoplasmic STAT3 impairs autophagy by interacting with forehead boxes 1 and 3 (FOXO1/FOXO3) (Oh et al., 2012). RHEB, one of the essential components of the mTOR complex, was previously reported to be an inhibitor of autophagy (Sciarretta et al., 2012). The role of STAT5B in autophagy remains obscure, whereas ATG4D and ATG5 were widely identified as inducers of autophagy (Betin and Lane, 2009; Agrotis et al., 2019; Zheng et al., 2019). As shown in Figure 5, the overexpression of DANCR led to the enhancement of STAT3, STAT5B, RHEB, ATG4D, and ATG5 by targeting miR-1301-3p/miR-5194. Although these five genes fine-tune autophagy in particular or even in opposite ways, excess DANCR appears to benefit autophagic flux in general. Therefore, we hypothesized that DANCR regulated autophagy through a network of gene interactions, and the resulting force of this network was positive overall. In other words, we presumed that ATG4D/ATG5 played a stronger role than STAT3/RHEB in the case of DANCR-mediated autophagy.

The dynamic process of autophagy is modulated by multiple genes. A growing number of studies have shown that the core regulator of autophagy is the ATG family, where each member of the family controls autophagy at different stages. For example, mouse ATG4b is in charge of either the lipidation or degreasing of LC3-II and then affects the coupling between LC3 protein and ATG12 protein (Tanida et al., 2004). Similarly, silencing ATG4D leads to an increase in the extracellular matrix and affects the smooth progression of the autophagy process (Andaloussi et al., 2017). Finally, mice lacking the ATG5 gene in monocytes were found to be more vulnerable to *M. tuberculosis* infection and died within 30 days in one study (Kimmey et al., 2015). In our study, we found that miR-1301-3p and miR-5194 inhibited autophagy by impairing ATG4D and ATG5. To the best of our knowledge, post-transcriptional regulation contains a variety of biologic functions, such as the splice and process of mRNA, the mRNA transfer from the nucleus to the cytoplasm, and RNA editing or interference. MiRNAs usually play roles in regulating the stability of mRNA and its degradation process. The pre-miRNAs enter the cytoplasm and are then matured by the Dicer enzyme. In addition, research has proven that miRNAs also play roles in translation. In most cases, to mediate the stability of mRNA, miRNAs bind to 3’UTR of the targets and then form an RNA-induced silencing complex (RISC) to degrade mRNAs. In this case, the amount of target mRNA will descend. Through ceRNAs, DANCR reverted the suppression caused by miR-1301-3p and miR-5194, which further proves the existence of the lncRNA–miRNA–mRNA axis *in vivo*. This finding was also confirmed by other scientists. The overexpression of DANCR inhibited the function of miR-6324, thereby enhancing the autophagy level of cardiomyocytes and protecting them from endoplasmic reticulum stress injury (Li et al., 2021). The accumulation of DANCR attenuated the anti-tumor effect of miR-758-3p in breast cancer cells. Silencing DANCR could lead to the inhibition of the malignant proliferation of breast cancer cells and the promotion of cell apoptosis and autophagy (Zhang et al., 2020). Other studies have pointed out that DANCR induces the proliferation, colony formation, and autophagy of hepatoma cells by increasing ATG7 and inhibiting miR-222-3p (Wang et al., 2020). Because miRNAs possess multiple targets, the function of sponging lncRNAs becomes even more complex. Thus, to clarify the function of lncRNAs, it is sensible to discuss their roles on a case-by-case basis.

Examples of such case-by-case situations in our study include the overexpression of DANCR leading to an improvement in the expression of the RHEB gene, which was a core component of the mTOR complex, as previously mentioned. mTOR is a negative regulator of autophagy. Studies have shown that miR-142-3p and miR-155 negatively regulate the expression of RHEB, inhibit the activity of the mTOR complex, and activate autophagy (Wang et al., 2013; Zhang et al., 2020). However, in our experiments, DANCR induced the expression of ATG5 and RHEB simultaneously by competitively adsorbing miR-1301-3p. It was found that the promoting effect of DANCR on autophagy was not simply regulated by one single gene but rather an output from a combination of various genes. Among these genes, both elements that promoted autophagy and inhibited factors of autophagy were present. Based on existing results, we considered ATG family members as leading players of all the above-mentioned genes in our experiments. To summarize, our data elucidated the abnormal increase of DANCR in tuberculosis patients. In addition, DANCR promoted autophagy by competitively sponging miR-1301-3p/miR-5194 and thus restrained the survival of intracellular *M. tuberculosis*. As shown in Figure 6, we used LV-DANCR THP-1 cell lines to perform CFU assays. It is clear that DANCR decreases the CFU of *M. tuberculosis* at 24 h. However, the intracellular bacilli are not thoroughly eliminated, and these remnants of bacteria might replicate in the next hours. Theoretically, the LV-DANCR cells that we constructed had the ability to overexpress DANCR for a long time. In other words, these cells were more resilient to infection in our case, but there were still lots of points to explore. For example, we think it is of great interest to assess if the regulation shown by DANCR is dependent on time. To draw the curve of autophagy intensity, autophagic flux monitoring may be an available solution.

## 5. Conclusion

Altogether, our data indicated that DANCR facilitated the expression of multiple genes *via* sponging miR-1301-3p and miR-5194. Although these genes regulated autophagy in diverse ways, the overall role of DANCR was positive on both autophagosome formation and fusion of autolysosomes, in addition to the restriction of intracellular H37Ra survival.

## 6. Statistical analysis

The results are presented as mean ± standard deviation (SD) of independent experiments. Statistical analyses were performed by using a two-tailed Student’s *t*-test. Significant differences were assigned to value of *p*s of <0.05, <0.01, <0.001, and < 0.0001, denoted by *, **,***, and ****, respectively.

## Data availability statement

The datasets presented in this study can be found in online repositories. The names of the repository/repositories and accession number(s) can be found in the article/supplementary material.

## Ethics statement

The studies involving human participants were reviewed and approved by ethics committee of Ningxia Medical University. The patients/participants provided their written informed consent to participate in this study.

## Author contributions

YQ: conceptualization, methodology, writing — original draft, and investigation. DJ: methodology, software, and writing — review and editing. ML, HW, TX, HZ, WS, and MH: investigation. GX: supervision, project administration, and funding acquisition. All authors contributed to the article and approved the submitted version.

## Funding

The study was funded by the Dongguan Science and Technology of Social Development Program (20221800905602), the National Natural Science Foundation of China (Nos. 82260321 and 81860355) and Research funding project of Ningxia Medical University (XT2022001).

## Conflict of interest

The authors declare that the research was conducted in the absence of any commercial or financial relationships that could be construed as a potential conflict of interest.

## Publisher’s note

All claims expressed in this article are solely those of the authors and do not necessarily represent those of their affiliated organizations, or those of the publisher, the editors and the reviewers. Any product that may be evaluated in this article, or claim that may be made by its manufacturer, is not guaranteed or endorsed by the publisher.

## Footnotes

^1^ www.mirdb.org

^2^ http://www.mirbase.org

^3^ http://mirwalk.umm.uni-heidelberg.de

## References

[ref1] Agrotis A. Pengo N. Burden J. J. Ketteler R. (2019). Redundancy of human ATG4 protease isoforms in autophagy and LC3/GABARAP processing revealed in cells. Autophagy 15, 976–997. doi: 10.1080/15548627.2019.1569925, PMID: 30661429 PMC6526816

[ref2] Andaloussi A. E. Habib S. Soylemes G. Laknaur A. Elhusseini H. Al-Hendy A.. (2017). Defective expression of ATG4D abrogates autophagy and promotes growth in human uterine fibroids. Cell Death Discov. 3:17041. doi: 10.1038/cddiscovery.2017.41, PMID: 28815060 PMC5554887

[ref3] Bai W. Liu H. Ji Q. Zhou Y. Liang L. Zheng R.. (2014). TLR3 regulates mycobacterial RNA-induced IL-10 production through the PI3K/AKT signaling pathway. Cell. Signal. 26, 942–950. doi: 10.1016/j.cellsig.2014.01.015, PMID: 24462705

[ref4] Betin V. M. Lane J. D. (2009). Atg4D at the interface between autophagy and apoptosis. Autophagy 5, 1057–1059. doi: 10.4161/auto.5.7.9684, PMID: 19713737

[ref5] Deretic V. Saitoh T. Akira S. (2013). Autophagy in infection, inflammation and immunity. Nat. Rev. Immunol. 13, 722–737. doi: 10.1038/nri3532, PMID: 24064518 PMC5340150

[ref6] Ehrt S. Schnappinger D. (2009). Mycobacterial survival strategies in the phagosome: defence against host stresses. Cell. Microbiol. 11, 1170–1178. doi: 10.1111/j.1462-5822.2009.01335.x, PMID: 19438516 PMC3170014

[ref7] Fratti R. A. Chua J. Vergne I. Deretic V. (2003). Mycobacterium tuberculosis glycosylated phosphatidylinositol causes phagosome maturation arrest. Proc. Natl. Acad. Sci. U. S. A. 100, 5437–5442. doi: 10.1073/pnas.0737613100 12702770 PMC154363

[ref8] Galluzzi L. Green D. R. (2019). Autophagy-independent functions of the autophagy machinery. Cells 177, 1682–1699. doi: 10.1016/j.cell.2019.05.026, PMID: 31199916 PMC7173070

[ref9] Gopalakrishnan A. Dietzold J. Verma S. Bhagavathula M. Salgame P. (2019). Toll-like receptor 2 prevents neutrophil-driven immunopathology during infection with mycobacterium tuberculosis by curtailing CXCL5 production. Infect. Immun. 87:e00760-18. doi: 10.1128/IAI.00760-18, PMID: 30559223 PMC6386542

[ref10] He J. Ou Q. Liu C. Shi L. Zhao C. Xu Y.. (2017). Differential expression of long non-coding RNAs in patients with tuberculosis infection. Tuberculosis (Edinb.) 107, 73–79. doi: 10.1016/j.tube.2017.08.007, PMID: 29050775

[ref11] Huang S. Huang Z. Luo Q. Qing C. (2018). The expression of lncRNA NEAT1 in human tuberculosis and its antituberculosis effect. Biomed. Res. Int. 2018:9529072. doi: 10.1155/2018/9529072 30534569 PMC6252192

[ref12] Jiang F. Lou J. Zheng X. M. Yang X. Y. (2021). LncRNA MIAT regulates autophagy and apoptosis of macrophage infected by mycobacterium tuberculosis through the miR-665/ULK1 signaling axis. Mol. Immunol. 139, 42–49. doi: 10.1016/j.molimm.2021.07.023, PMID: 34454184

[ref13] Khan S. R. Manialawy Y. Siraki A. G. (2019). Isoniazid and host immune system interactions: a proposal for a novel comprehensive mode of action. Br. J. Pharmacol. 176, 4599–4608. doi: 10.1111/bph.14867, PMID: 31517993 PMC6965675

[ref14] Kimmey J. M. Huynh J. P. Weiss L. A. Park S. Kambal A. Debnath J.. (2015). Unique role for ATG5 in neutrophil-mediated immunopathology during *M. tuberculosis* infection. Nature 528, 565–569. doi: 10.1038/nature16451, PMID: 26649827 PMC4842313

[ref15] Lai Y. F. Lin T. M. Wang C. H. Su P. Y. Wu J. T. Lin M. C.. (2016). Functional polymorphisms of the TLR7 and TLR8 genes contribute to mycobacterium tuberculosis infection. Tuberculosis (Edinb.) 98, 125–131. doi: 10.1016/j.tube.2016.03.008, PMID: 27156628

[ref16] Li J. Xie J. Wang Y. Z. Gan Y. R. Wei L. Ding G. W.. (2021). Over-expression of lncRNA Dancr inhibits apoptosis and enhances autophagy to protect cardiomyocytes from endoplasmic reticulum stress injury via sponging microRNA-6324. Mol. Med. Rep. 23:116. doi: 10.3892/mmr.2020.11755 33300079 PMC7723073

[ref17] Liu Y. Li J. Y. Chen S. T. Huang H. R. Cai H. (2016). The rLrp of mycobacterium tuberculosis inhibits proinflammatory cytokine production and downregulates APC function in mouse macrophages via a TLR2-mediated PI3K/Akt pathway activation-dependent mechanism. Cell. Mol. Immunol. 13, 729–745. doi: 10.1038/cmi.2015.58, PMID: 26166760 PMC5101441

[ref18] Liu G. Xia R. Wang Q. Wang Z. Ying B. Yan H. (2020). Significance of LncRNA CASC8 genetic polymorphisms on the tuberculosis susceptibility in Chinese population. J. Clin. Lab. Anal. 34:e23234. doi: 10.1002/jcla.23234 32034808 PMC7307370

[ref19] Liu Z. Z. Zhang C. Y. Huang L. L. Liu W. (2019). Elevated expression of lncRNA SNHG15 in spinal tuberculosis: preliminary results. Eur. Rev. Med. Pharmacol. Sci. 23, 9017–9024. doi: 10.26355/eurrev_201910_19303, PMID: 31696491

[ref20] Mercer T. R. Dinger M. E. Mattick J. S. (2009). Long non-coding RNAs: insights into functions. Nat. Rev. Genet. 10, 155–159. doi: 10.1038/nrg2521 19188922

[ref21] Nakamura S. Yoshimori T. (2017). New insights into autophagosome-lysosome fusion. J. Cell Sci. 130, 1209–1216. doi: 10.1242/jcs.196352, PMID: 28302910

[ref22] Oh H. M. Yu C. R. Dambuza I. Marrero B. Egwuagu C. E. (2012). STAT3 protein interacts with class O Forkhead transcription factors in the cytoplasm and regulates nuclear/cytoplasmic localization of FoxO1 and FoxO3a proteins in CD4(+) T cells. J. Biol. Chem. 287, 30436–30443. doi: 10.1074/jbc.M112.359661, PMID: 22761423 PMC3436293

[ref23] Ouimet M. Koster S. Sakowski E. Ramkhelawon B. van Solingen C. Oldebeken S.. (2016). Mycobacterium tuberculosis induces the miR-33 locus to reprogram autophagy and host lipid metabolism. Nat. Immunol. 17, 677–686. doi: 10.1038/ni.3434, PMID: 27089382 PMC4873392

[ref24] Pan Z. Wu C. Li Y. Li H. An Y. Wang G.. (2020). LncRNA DANCR silence inhibits SOX5-medicated progression and autophagy in osteosarcoma via regulating miR-216a-5p. Biomed. Pharmacother. 122:109707. doi: 10.1016/j.biopha.2019.109707 31918278

[ref25] Rohini K. Srikumar P. S. (2013). Insights from the docking and molecular dynamics simulation of the Phosphopantetheinyl transferase (PptT) structural model from mycobacterium tuberculosis. Bioinformation 9, 685–689. doi: 10.6026/97320630009685, PMID: 23930020 PMC3732441

[ref26] Sciarretta S. Zhai P. Shao D. Maejima Y. Robbins J. Volpe M.. (2012). Rheb is a critical regulator of autophagy during myocardial ischemia: pathophysiological implications in obesity and metabolic syndrome. Circulation 125, 1134–1146. doi: 10.1161/CIRCULATIONAHA.111.078212, PMID: 22294621 PMC3337789

[ref27] Shen S. Niso-Santano M. Adjemian S. Takehara T. Malik S. A. Minoux H.. (2012). Cytoplasmic STAT3 represses autophagy by inhibiting PKR activity. Mol. Cell 48, 667–680. doi: 10.1016/j.molcel.2012.09.013, PMID: 23084476

[ref28] Siqueira M. D. S. Ribeiro R. M. Travassos L. H. (2018). Autophagy and its interaction with intracellular bacterial pathogens. Front. Immunol. 9:935. doi: 10.3389/fimmu.2018.00935, PMID: 29875765 PMC5974045

[ref29] Sun W. Zu Y. Fu X. Deng Y. (2017). Knockdown of lncRNA-XIST enhances the chemosensitivity of NSCLC cells via suppression of autophagy. Oncol. Rep. 38, 3347–3354. doi: 10.3892/or.2017.6056, PMID: 29130102 PMC5783579

[ref30] Tanida I. Ueno T. Kominami E. (2004). LC3 conjugation system in mammalian autophagy. Int. J. Biochem. Cell Biol. 36, 2503–2518. doi: 10.1016/j.biocel.2004.05.009, PMID: 15325588 PMC7129593

[ref31] Tong X. Gu P. C. Xu S. Z. Lin X. J. (2015). Long non-coding RNA-DANCR in human circulating monocytes: a potential biomarker associated with postmenopausal osteoporosis. Biosci. Biotechnol. Biochem. 79, 732–737. doi: 10.1080/09168451.2014.998617, PMID: 25660720

[ref32] Wang X. Cheng M. L. Gong Y. Ma W. J. Li B. Jiang Y. Z. (2020). LncRNA DANCR promotes ATG7 expression to accelerate hepatocellular carcinoma cell proliferation and autophagy by sponging miR-222-3p. Eur. Rev. Med. Pharmacol. Sci. 24, 8778–8787. doi: 10.26355/eurrev_202009_22816, PMID: 32964966

[ref33] Wang Y. Li Z. Xu S. Li W. Chen M. Jiang M.. (2022). LncRNA FIRRE functions as a tumor promoter by interaction with PTBP1 to stabilize BECN1 mRNA and facilitate autophagy. Cell Death Dis. 13:98. doi: 10.1038/s41419-022-04509-1, PMID: 35110535 PMC8811066

[ref34] Wang C. Z. Yan G. X. Dong D. S. Xin H. Liu Z. Y. (2019). LncRNA-ATB promotes autophagy by activating yes-associated protein and inducing autophagy-related protein 5 expression in hepatocellular carcinoma. World J. Gastroenterol. 25, 5310–5322. doi: 10.3748/wjg.v25.i35.5310, PMID: 31558875 PMC6761242

[ref35] Wang J. Yang K. Zhou L. Minhaowu Y. Wu M. Zhu X.. (2013). MicroRNA-155 promotes autophagy to eliminate intracellular mycobacteria by targeting Rheb. PLoS Pathog. 9:e1003697. doi: 10.1371/journal.ppat.1003697, PMID: 24130493 PMC3795043

[ref36] Xu T. Xu X. Chu Y. Jiang D. Xu G. (2021). Long-chain non-coding RNA GAS5 promotes cell autophagy by modulating the miR-181c-5p/ATG5 and miR-1192/ATG12 axes. Int. J. Mol. Med. 48. doi: 10.3892/ijmm.2021.5042, PMID: 34608496 PMC8510682

[ref37] Xu Y. Yu J. Ma C. Gong Z. Wu X. Deng G. (2021). Impact of knockdown LincRNA-Cox2 on apoptosis of macrophage infected with bacillus Calmette-Guérin. Mol. Immunol. 130, 85–95. doi: 10.1016/j.molimm.2020.11.008, PMID: 33250268

[ref38] Yao L. Yang L. Song H. Liu T. G. Yan H. (2020). Silencing of lncRNA XIST suppresses proliferation and autophagy and enhances vincristine sensitivity in retinoblastoma cells by sponging miR-204-5p. Eur. Rev. Med. Pharmacol. Sci. 24, 3526–3537. doi: 10.26355/eurrev_202004_20812, PMID: 32329826

[ref39] Zhang Y. Gao M. Chen L. Zhou L. Bian S. Lv Y. (2020). Licochalcone a restrains microphthalmia-associated transcription factor expression and growth by activating autophagy in melanoma cells via miR-142-3p/Rheb/mTOR pathway. Phytother. Res. 34, 349–358. doi: 10.1002/ptr.6525, PMID: 31793097

[ref40] Zhang X. H. Li B. F. Ding J. Shi L. Ren H. M. Liu K.. (2020). LncRNA DANCR-miR-758-3p-PAX6 molecular network regulates apoptosis and autophagy of breast cancer cells. Cancer Manag. Res. 12, 4073–4084. doi: 10.2147/CMAR.S254069, PMID: 32581581 PMC7269637

[ref41] Zhang H. Liu L. Chen L. Liu H. Ren S. Tao Y. (2021). Long noncoding RNA DANCR confers cytarabine resistance in acute myeloid leukemia by activating autophagy via the miR-874-3P/ATG16L1 axis. Mol. Oncol. 15, 1203–1216. doi: 10.1002/1878-0261.12661, PMID: 33638615 PMC8024725

[ref42] Zheng W. Xie W. Yin D. Luo R. Liu M. Guo F. (2019). ATG5 and ATG7 induced autophagy interplays with UPR via PERK signaling. Cell Commun. Signal. 17:42. doi: 10.1186/s12964-019-0353-3, PMID: 31060556 PMC6503447

